# Clinical Hypnosis with Children and Adolescents—What? Why? How?: Origins, Applications, and Efficacy

**DOI:** 10.3390/children1020074

**Published:** 2014-08-12

**Authors:** Daniel P. Kohen, Pamela Kaiser

**Affiliations:** 1 National Pediatric Hypnosis Training Institute (NPHTI), Developmental-Behavioral Pediatrics, Partners-in-Healing of Minneapolis, 10505 Wayzata Boulevard, Suite #200, Minnetonka, MN 55305, USA; 2 National Pediatric Hypnosis Training Institute (NPHTI), Private Practice, 1220 University Drive, Suite #104, Menlo Park, CA 94025, USA; E-Mail: drpkaiser@gmail.com

**Keywords:** hypnosis, pediatric hypnosis, self-regulation, pediatric skills development, self-hypnosis, NPHTI, pediatric hypnosis workshops

## Abstract

This review article addresses the process, intention, and therapeutic value of clinical hypnosis with children and adolescents. A brief historical perspective is followed by a digest of the published laboratory and clinical research that has accelerated substantially over the past two decades. This review lends appropriate credence to the benefits and integration to clinical practice of this powerful tool for teaching young people self-regulation skills. The breadth of application is described, and several clinical vignettes are provided as examples of what is possible. In addition to the provision of the most relevant citations in the pediatric, psychological, and neuroscience literature, this synopsis concludes with information regarding availability of skill development training in pediatric clinical hypnosis.

## 1. Introduction

The review that follows is designed to be a panoramic snapshot of the current state of the art, science, and clinical practice of child hypnosis and hypnotherapy. While substantial reference resource material is provided and clinical examples described, this is nonetheless a synopsis, and readers are advised and strongly encouraged to consult the resources and to avail themselves of “hands-on” skill-development workshop training to develop skills necessary to begin helping children with clinical hypnosis.

The field of pediatric hypnosis has blossomed in many ways and directions over time:

- From a backdrop focus on societal and cultural beliefs and norms to a context of fostering respect for the child’s creativity, imagination, unique perceptions, and choices.
- From the view of hypnosis as a ritual completed by an authority figure *on* the individual to an interactive, dynamic “dance-like” experience *between* two individuals, with the clinician sometimes “leading” and other times “pacing” while the child leads.
- From the use, writings, and research by a select group of clinicians, *i.e*., physicians and psychologists, to the broadened utilization by and contributions from various types of health care providers with advanced degrees.
- From apprenticeship learning at the bedside of highly specified hypnotic techniques to the richness of multi-modal and multi-sensory training within a group setting, based on research about adult learning and skill acquisition, emphasizing the multiple paths toward similar clinical goals.
- From definitions based on the fixed views and narrow theoretical concepts of a few to the fertile exchange by many within a global community.
- From precise suggested commands applied rigidly to *all* patients to open-ended, permissive, and *individualized* suggestions utilizing the specific needs, resources, and interests of each child or teen.
- From making conclusions and generalizations about the hypnotic experience based solely on behavioral observation to incorporating double-blind research studies evaluated in Cochrane reviews and state-of-the-art neuroimaging to correlate with the varying types of hypnotic behavior.

## 2. Brief History of Hypnosis & Hypnosis with Children

In ancient times hypnotic-like techniques were utilized in healing and other ceremonies [1]. (Biblical references in both the Old and New Testaments describe accounts of ill children responding to healing methods based on faith and suggestion (I Kings XVII: 17–24; Mark IX: 17–27). Primitive cultures have employed trance phenomena with children in initiation rites and other ceremonies [1].

The modern history of hypnosis—in both children and adults—can be found in the rich, fascinating, and challenging history of many physicians of 18th century Europe and even more so in the 19th century. Many physicians contributed substantial clinical experience and theoretical concepts to our current understanding of clinical hypnosis and its usefulness in clinical health care. Amongst many, these included Franz Anton Mesmer (1734–1815), the British Surgeon John Elliotson (1791–1868) who also introduced the stethoscope to Great Britain, James Braid (1795–1860) who coined the word hypnosis, and Jean-Martin Charcot (1835–1893) whose descriptions of hypnosis in neurological terms gave it a new measure of scientific respectability. Of import, Liebault (1823–1904) and Bernheim (1840–1919) founded the School of Nancy devoted to the scientific investigation of hypnosis and described the first research on children’s hypnotic susceptibility, describing over 750 subjects and the peak “susceptibility” thought in those days to occur between the ages of 7 and 14 years [2] (Kohen and Olness, 2011; pp. 7–12).

A more detailed description of the early history of hypnosis with children is beyond the scope of this article and can be found in Kohen and Olness’ text Hypnosis and Hypnotherapy with Children (4th edition, 2011) [2].

Kohen and Olness (2011) note the apparent beginning of child hypnosis in the U.S. [2] with Baldwin’s paper entitled “Suggestion in Infancy” in *Science* published in 1891 [3]. By 1900, interest in child hypnosis in Europe and the U.S. had decreased and it was not until the late 1950s and early 1960s that there was a resurgence of interest in research in Child Hypnosis. In the late 1950s psychiatrists Dr. Milton Erickson [4] and Dr. M. Erik Wright [5] described their clinical use of hypnosis/hypnotherapy with children.

In the 1960s Dr. Franz Baumann, a general pediatrician in San Francisco, was the first pediatrician to become President of a major Hypnosis Society (The American Society of Clinical Hypnosis). He published papers on the applications of hypnosis for adolescent drug abuse, enuresis, and encopresis [6,7]. In that decade several so-called Susceptibility Scales for Hypnotic Susceptibility in Children were developed (London and Cooper, 1969, [8]; Barber & Calverley, 1963, [9]). The “gold standard” for hypnotic susceptibility in children, the Stanford Children’s Hypnotic Susceptibility Scale (used primarily in research) was developed and published in 1979 by Arlene Morgan, Ph.D. & Josephine Hilgard, Ph.D. (see Kohen and Olness, 2011, appendix) [2].

Additional clinical reports emerged in the 1970s, most notably by G. Gail Gardner, Ph.D. [10], Karen Olness, M.D., the acknowledged pioneer and “mother of pediatric clinical hypnosis” [11], and Lonnie Zeltzer, M.D. and Sam LeBaron, M.D, Ph.D. who did early and important clinical and research work with pediatric oncology patients [12,13,14], The first formal workshop in child hypnosis was taught in 1976 at the International Congress of Hypnosis in Philadelphia [15].

The first textbook, Hypnosis and Hypnotherapy with Children, was written by G. Gail Gardner, Ph.D. and Karen Olness, M.D. and published in 1981 [16]. The second edition was published in 1988 [17], the third in 1996 by Karen Olness, M.D. and Daniel P. Kohen, M.D. [18], and the most recent, fourth edition in 2011 by Kohen and Olness [2]. The third edition was also translated into and published in German [19] and French [20].

Beyond milestones noted in Table 1 [21,22,23,24,25,26,27,28,29,30,31,32], many clinical applications of hypnosis in child health began to be reported in the 1980s and increasingly in the 1990s, notably clinical and research papers on the efficacy and applications for clinical hypnosis for children with asthma [33,34], children with cancer [14,22,23,24,25] and tic and Tourette Syndrome [35].

Following the aforementioned initial pediatric clinical hypnosis training workshop in 1976, occasional half-day to two day advanced workshops in pediatric hypnosis were offered to experienced child health clinicians sporadically during annual conferences of the American Society of Clinical Hypnosis (ASCH) and the Society for Clinical and Experimental Hypnosis (SCEH). Beginning in 1987, annual 3-day pediatric hypnosis workshops were offered under the auspices of the American organization, the Society for Developmental-Behavioral Pediatrics (SDBP), as part of its annual meeting. Given this workshop’s wonderful success the following year concurrent Introductory *and* Intermediate Level training workshops were offered, and an Advanced Pediatric Clinical Hypnosis Workshop was added the next year. These three concurrent workshops were provided annually through 2009. Beginning in 2010, the Faculty from these preceding 24 years remained together and the National Pediatric Hypnosis Training Institute (NPHTI) was co-founded by Daniel Kohen, MD and Pamela Kaiser, PhD, CPNP, CNS. By Fall, 2014, NPHTI’s 5th annual tri-level workshops will have tripled their faculty in order to meet the growing attendance by multi-disciplinary clinicians from around the globe.

**Table 1:** Key Milestones in 20th–21st Century Evolution of Pediatric Hypnosis.

Year	Milestone	Comment/Reference
1984	Largest clinical series (to date) of Pediatric patients treated with Hypnosis	[21] Kohen, D.P., Olness, K.N., Colwell, S., & Heimel
1986 & 1999	Seminal Videos produced documenting the value of hypnosis in children with Cancer: “No Fears, No Tears” and the sequel “No Fears, No Tears- 13 years later”	[22,23] Leora Kuttner, Ph.D. Vancouver, BC Canada [24,25]
1987 (to 2009)	First Annual Workshops in Pediatric Clinical Hypnosis for Child Health Clinicians–under auspices of Society for Developmental and Behavioral Pediatrics	[2] Inaugural in Disneyland 1987 under auspices of SDBP Annually 1987–2009.
1989	First Pediatric Psychoneuroimmunology study	[26]
1997	“Imaginative Medicine – Hypnosis in Pediatric Practice “Video documenting value of hypnosis in primary care	[27] Laurence I. Sugarman, M.D. Rochester Institute of Technology, Rochester NY
2005, 2009	Harry the Hypno-potamus: Metaphorical Tales for Pediatric Problems (Vol I 2005, Vol II in 2009)	[28,29] Linda Thomson, CPNP
1996, 2011	Hypnosis and Hypnotherapy with Children published 3rd edition 1996, 4th edition 2011	[2,18] 3rd ed Olness KN and Kohen, DP 1996 4th ed Kohen, DP and Olness, KN 2011
2007—2nd ed 2013	Therapeutic Hypnosis with Children and Adolescents published 1st edition 2007 and 2nd edition 2014	[30] Editors: WC Wester and LI Sugarman 2007 2nd ed: Sugarman & Wester 2014
2010 (annual)	**Formation of NPHTI—National Pediatric Hypnosis Training Institute** -Inaugural Annual Skill Development Workshops in Pediatric Clinical Hypnosis (Introductory, Intermediate and Advanced) **www.nphti.com**	[31] Co-Founders and Co-Directors Pamela Kaiser, PhD, CPNP, CNS Daniel P. Kohen, MD, FAAP, ABMH 2010–2014 and ongoing **www.nphti.com**
2010	“Non-pharmacological treatment of tics in Tourette Syndrome with videotape training in self-hypnosis.” ***JDBP***	[2] (p 310) Lazarus and Klein article including link to video illustration of hypnosis with a child with Tourette Syndrome.
1990, 1992, 1997, 2002, 2005, 2009, 2013	Kindertagung = Largest Child Hypnosis Congress *in the world*—begun 1990, most recent Heidelberg Germany, 2013	[32] Bernhard Trenkle, Dipl Psych

Kindertagung—the Child Hypnosis Congress—is the largest child hypnosis congress in the world, due to the vision of organizer and teacher, Bernhard Trenkle, Dipl. Psych. and Director of the Milton Erickson Institute in Rottweil, Germany. It was established initially in 1990 in Rottweil, and attracted 300 participants. Two years later, it was offered by a larger faculty in Heidelberg, Germany, and over 450 attended. Over the next sixteen years, it was offered four additional times with continued exponential growth in attendance. In 2013 the 7th Kindertagung was held in Heidelberg , with 2000 attendees including 114 faculty from Germany, France, Italy, Switzerland, South Africa, Canada, and the U.S. [32].

## 3. Definition

Over the past three decades professionals have proposed a variety of theoretical definitions based upon controlled clinical laboratory research [36].

The American Society of Clinical Hypnosis provides the following definition on its website (www.asch.net):

“**Definition of Hypnosis:** Hypnosis is a state of inner absorption, concentration and focused attention. It is like using a magnifying glass to focus the rays of the sun and make them more powerful. Similarly, when our minds are concentrated and focused, we are able to use our minds more powerfully. Because hypnosis allows people to use more of their potential, learning self-hypnosis is the ultimate act of self-control…. Recent research supports the view that hypnotic communication and suggestions effectively changes aspects of the persons physiological and neurological functions.”

The American Psychological Association Division 30 states in their 2014 revised official definition [37].

**Hypnosis** is a state of consciousness involving focused attention and reduced peripheral awareness characterized by an enhanced capacity for response to suggestion; **hypnotizability** is an individual’s ability to experience suggested alterations in physiology, sensations, emotions, thoughts, or behavior during hypnosis; and **hypnotherapy**: The use of hypnosis in the treatment of a medical or psychological disorder or concern.

OR, as pediatrician Laurence Sugarman added his perspective [38] (Sugarman, NPHTI presentation, September 2013).

“When we are IN hypnosis, we intensify our attention, decrease our peripheral awareness and become more receptive to new ideas and associations whenever we reinforce, rewire, reframe or otherwise alter the neurophysiological networks we call “experience”. Trance is what happens when we engage in changing our minds.” He goes on to say that “Hypnosis is about creating therapeutic interpersonal space for trance-formation that leads to transformation.” [38] and, provides a definition that says that “Hypnosis is A skill set involving interpersonal communication designed to facilitate therapeutic change in maladaptive psycho- physiological reflexes.”

[38] (Otherwise, of course, WHY do it?!)

More recently theories and definitions have been proposed based upon neurophysiological research aimed precisely at identifying and describing the neurophysiologic correlates of hypnotic behavior, reported and observed hypnotic features and effects, now verified with the availability and use of functional MRI (fMRI), PET scans, and related physiologic measures [39,40] *etc*.

Using brain imaging, cognitive neuroscientists have identified the power that suggestions have over attention functions and associated brain networks and their impact on physical and mental experience (Raz, 2011, [40]). Still, Amir Raz, a leader in the field of neurohypnosis, cautions about the common over-interpretation of fMRI findings. He notes that researchers are now using newer techniques combined with fMRIs in the goal of identifying brain regions associated with different cognitive functions [40] We await further exciting discoveries from these and other researchers.

The most recent issue of Mind-Body-Regulation [41] (MBR, 5 May 2014) brought together the world’s most distinguished scholars to create a decisive definition of hypnosis that has eluded scholars over the past century. While they provided stimulating food for thought, they did not reach a mutually agreed upon conclusion or provide a unifying definition [41].

Although there is no universally agreed-upon or scientifically validated definition of hypnosis, we prefer a descriptive definition that elucidates what we believe to be the fundamental ingredients of the hypnotic experience and phenomenon from a clinical perspective/in a clinical setting:

A spontaneously occurring or induced alternative state of awareness (with or without relaxation, which may or may not be evident) in which an individual develops a focused concentration on some idea or image with the expressed purpose of maximizing potential, creating a CHANGE, and/or reducing or resolving some problem.

[2] (Kohen and Olness, 2011)

## 4. What is It?

Building on the preceding purposes delineated in the definition by Kohen and Olness, we would like to expand our conceptualization of clinical pediatric hypnosis as a tool to facilitate the evolution and refinement of **self-regulation skills and capacities**. More specifically, hypnosis is a tool to strengthen children’s existing or under-developed skills in self-regulation, *i.e*., capacities to *shift attention*, maintain *focused attention*, inhibit and control reflexive actions, delay gratification, use problem solving strategies, and self-monitor and modulate thinking, emotion, behavior, and psycho-physiological reactivity [42]. Children typically develop this capacity during infancy and childhood [43]. With appropriate clinical pediatric hypnosis training, child health clinicians can help children and youth to identify, discover, develop, and cultivate these capacities and other internal resources for their own benefit in managing and shaping their responses to a broad range of health and life problems, dilemmas, and challenges [42]. In this regard clinical hypnosis helps children and their parents develop and refine the concept of “skills not pills” [44] as strategies to apply to their particular problems or concerns.

We wish to emphasize that we believe that the critical ingredients of the therapeutic hypnotic experience in children (as well as in adults) are focused attention to and absorption in imagination which includes a focus on multi-sensory imagery and a particular ***cultivation of that imagination*** on steps and strategies toward goals for resolution of problems and change of ineffective patterns. Examples of possible specific goals include diminution or disappearance of undesirable symptoms, accurate discrimination of distorted thinking about situations and stressors, re-framing of perception of problems as solvable and conditions as manageable, building positive expectations, control of exaggerated reactivity, and creation and enhancement of the belief in the ability of the mind and body to work together to evoke and create desirable changes in outcome.

While this is not the forum for an expanded discussion of the similarities and differences between hypnosis and other self-regulatory activities, we are aware of other modalities that incorporate experiential learning and mechanisms for change. We are clear in telling our clients and patients that “some people call this work hypnosis, some call it imagery, some call it visualization, others refer to it as mindfulness, and some call it biofeedback.” We indicate our belief that these approaches bear some similarity to one another and may reflect some overlap of the same phenomenology. These activities share the goals of helping people to cultivate internal resources, to explore new possibilities, to accomplish physiologic, perceptual, sensory, and behavioral changes, and to facilitate mind-body communication and modulation [45,46,47].

## 5. Why Use Hypnosis? Why Do It?

In a video prepared by one of us (DK) for teaching clinicians how children understand and apply self-hypnosis [48] an eleven-year-old boy with Asthma who improved substantially with the application of his self-hypnosis skills is asked “**How** does **your** hypnosis help you with your asthma?” He begins by saying “How does it help the medicine work better and faster? Yeah, it does, I don’t know how. How does it help me feel better… breathe easier? Well, ummmmm, uhhh, it’s kind of hard to explain…..” and then turns his face to the camera and says, “Well, it works….. okay, all you people out there, it works!” This clinical evidence is followed below by a review of the published evidence.

## 6. Wide Spectrum of Applications

As described in the historical review, the beginning of a surge of clinical reports and clinical research in pediatric hypnosis began in the 1970s and continues today. This substantive increase may be causally related—and certainly temporally related—to the concurrent evolution of increased workshop training in pediatric clinical hypnosis during these decades as discussed above.

In 1984 Kohen and colleagues published what remains today the largest clinical series of children (505 children) and youth treated with hypnosis for a variety of clinical problems, including enuresis, pain, asthma, habit disorders, obesity, encopresis, and anxiety [21]. In 1987 Olness, Uden and McDonald published their seminal research documenting the superiority of hypnosis training to medication in the treatment of juvenile migraine [49].

In appropriate search of physiologic correlates of the hypnotic experience in children, studies have been conducted describing changes found in peripheral skin temperature during hypnosis [50], in transcutaneous oxygen flux [51], cardiovascular changes in association with hypnosis [52,53], pulmonary function changes in children with asthma learning hypnosis [34,54].

The value of hypnosis in modulating the negative effects of chemotherapy for children with cancer (such as nausea and vomiting) has been demonstrated in the research and clinical work of Zeltzer and LeBaron [12,13,14], Olness [55], Jacknow and colleagues [56], and dramatically in Kuttner’s award-winning educational videos “No Fears, No Tears” and “No Fears, No Tears—13 years Later” [22,23] 1986, and Making Every Moment Count [25]. Kohen and Zajac recently described the efficacy of hypnosis for headaches in children and teens [57]. Subsequently Kohen reported an important follow-up survey demonstrating the long-term benefits of hypnosis for those who had originally benefited from learning hypnosis for relief of headaches and reported not only long-term sustained relief, but also the application of those skills to other areas of their lives [58]. Recently, Sugarman and colleagues have published an award-winning article describing new theories and promising research regarding the value of hypnosis (and biofeedback) with young people with autism [59].

In recent years the increase in use of clinical hypnosis with children has resulted in a plethora of publications for a wide variety of clinical problems. For example, in preparation of the fourth edition of Hypnosis and Hypnotherapy with Children (2011) [2] the authors reviewed over 500 newly published articles from the English, French, and German literature, published since the third edition in 1996 [18].

For a detailed and wide-ranging description of the myriad of clinical conditions for which hypnosis is applicable and effective in children and youth, readers are encouraged to consult the two standard texts in this field, Kohen and Olness’ 2011 fourth edition Hypnosis the Hypnotherapy with Children [2] and Sugarman and Wester’s edited text, Therapeutic Hypnosis with Children and Adolescents, secondedition (2014) [30].

Additionally, we refer to the following Table 2 that highlights a broad range of applications.

**Table 2:** Applications of Hypnotherapy for Children and Adolescents *.

Category	Examples	Key References
1. Habit Problems	Thumb-sucking, nail-biting, Hair-pulling, Nocturnal Enuresis, Habit Cough	GGailGardner 1978 [10]
		D Kohen 1996 [60]
		L Thomson (2002) [61]
		Gottsegen (2003) [62]
		Anbar and Hall (2004) [63]
		Shenefelt (2004) [64]
		Anbar (2007) [65]
		Olness (1975) [11]
2. Mental Health Conditions	Separation Anxiety • Social Anxiety • Performance Anxiety (speeches, recital, sports) • post traumatic stress disorder (PTSD) • obsessive compulsive disorder (OCD) • Phobias Grief & Bereavement Major Depression • Dysthymia • Adjustment Disorder with Depression and Anxiety Anger • Adjustment Disorder • Coping with family change	Kaiser (2011) [42]
		Kaiser (2014) [94]
		Spies (1979) [95]
		Kuttner, Friedrichsdorf (2014) [96]
		Golden (2012) [97]
		Kerns et al (2013) [98]
		Yapko (2001) [99]
		Yapko (2006) [100]
		Yapko (2011) [70]
		Kohen, Murray (2006) [101]
		Kohen (2014) [102]
		Cyr, Culbert, Kaiser 2003 [66]
		Tschann, Kaiser, Boyce, Chesney, Alkon-Leonard (1996) [67]
		Culbert & R-Cyr, Ped Mind-Body Interface (2005) [68]
		Olness (2009) [69]
		Yapko (2011) [70]
3. Psycho-Physiological Disorders	Asthma • Migraine HA •IBS Inflammatory Bowel Disease • Warts • Tourette Syndrome	Kohen & Zajac 2007 [57]
		Kohen (2010, 2011) [58,71]
		Olness, MacDonald Uden (1987) [44]
		LaBaw (1975) [72]
		Anbar (2003) [73]
		Hackman ( 2000 ) [74]
		Kohen (1995) [33]
		Kohen, Wynne (1997) [34]
		Culbert, Kajander, Kohen Reaney (1996) [75]
		Gottsegen (2011) [76]
		Kuttner et al (2006) [77]
		Vlieger, Menko-Frankenhuis (2007) [78]
		Pendergrast (2003) [79]
		Lazarus, Klein (2010) [2] (p. 310)
		Kohen, Botts (1987) [35]
		Kohen (1995) [80]
4. Pain	Acute (injury, illness, medical procedures) • Chronic pain • Recurrent Pain	Kuttner (2006) [81]
		Olness (1981) [50]
		Berberich, Landman (2009) [82]
		Myers, Bergman, Zeltzer (2004) [83]
		Zeltzer, Dolgin, LeBaron, S., LeBaon, C. (1991) [84]
		Vlieger, Menko-Frankenhuis (2007) [78]
		Gottsegen (2011) [76]
		Anbar (2001) [85]
		Antich (1967) [86]
		Bernstein (1965) [87]
		Butler et al (2005) [88]
		French, GM et al (1994) [89]
		Kohen, (1996) [90]
		Kuttner (1988) [24]
		Kuttner (2010) [91]
		LeBaron, Hilgard (1984) [14]
		Tsao, *et al*., (2007) [92]
		Uman et al (2008) [93]
		Gulewitsch, Muller, Hutzinger, Schlarb (2013) [109]
5. Sleep Disorders	Sleep onset insomnia (Anx), Circadian Rhythm Disorder, Parasomnias	Culbert, Kajander (2007) [103]
		Kuttner (2009) [104]
		Kaiser (2011) [42]
		Kohen et al (1996) [105]
		Stanton (1989) [106]
		Schlarb, Liddle, Hautzinger (2011) [110]
6. Chronic Illness	Adjustment & coping Palliative Care, Grief, Bereavement	Anbar (2000) [54]
		Anbar (2001) [85]
		Zeltzer, Schlank (2005) [107]
		Kuttner, Friedrichsdorf (2014), [96]
		Kuttner (2006), [81]
		Kuttner (2003), [25]
		Kohen, Olness (2011, Chapter 15) [2]
		Gardner (1976) [108] *

* While several illustrative references are cited, additional relevant examples and references can be found in the two major textbooks, noted in references [2,30].

The value of brain imaging in amplifying how hypnotic suggestions impact mental and physical experience is described by Lifshitz, Cusumano, and Raz (2013) [111]. It is also noteworthy that Cochrane and other systematic reviews support the efficacy of pediatric hypnosis for various clinical applications, including Curtis *et al*., (2012) [112], Gold *et al*., (2007) [113], Landier & Tse (2010) [114], McClaffery (2011) [115], Rickardson *et al*., (2006) [116], Schnur *et al*., (2008) [117], and Uman *et al*., (2013) [118].

## 7. Case Presentations

The remainder of this paper offers several case vignettes to illustrate common applications of hypnosis and hypnotherapy in every-day child and adolescent health care. These case examples also demonstrate the nature and value of these empowering hypnosis strategies for children who previously struggled substantially before developing various self-regulation skills:

### 7.1. Case of Anxiety

#### 7.1.1. CASE #1: Performance Anxiety: From Baseball Bawling to Behavioral and Emotional Self-Regulation

J. is a 9-year-old fourth grader who presented with a long history of anxiety about not measuring up to his own very high standards. This was associated with poor emotional and behavioral self-regulation when frustrated and disappointed with his mistakes, which was particularly detrimental when playing his beloved sport, baseball. Despite his Dad’s coaching tricks to let go of these feelings, he typically needed to be assisted off the pitcher’s mound due to uncontrollable tears, kicking the dirt, and hitting himself with his glove. He was unable to “regroup” from this marked emotional and behavioral disintegration and the sobbing continued, resulting in reduced playing time, embarrassment, and later regret and further anxiety.

As with all initial encounters with new patients, developing rapport with the child and parents is critical to the process and outcome of the treatment. Similarly, the clinician learns the child’s personal and family history, and assesses motivation, expectations, strengths and other internal resources, such as capacity to accurately discriminate or interpret a situation and to self-regulate their emotions, thoughts, and behavior in other areas of their life. Time is spent to appraise the child’s understanding of the “problem” as well as *how* they accomplished past mastery experiences.

While there may be some exceptions, the more formalized introduction of hypnosis *per se* and discussion and demystification of hypnosis may not take place until subsequent visit(s). Given this child’s strong interest in sports, the clinician linked the explanation about hypnosis to references about top athletes’ learned skills in focused attention, mental rehearsal, and being “in the zone”. She emphasized that “learning to do ‘self-hypnosis’ like those athletes do will certainly help his game”.

Children in this age range are particularly receptive and responsive to hypnosis. The clinician capitalizes on this developmental phase, by utilizing the child’s expanded attention span, intellectual curiosity, motivation to master new skills, capacity to understand and create metaphors, and the ability to access memories of the past and to richly imagine the future.

This child saw his “hypnosis coach” weekly for several sessions, during which hypnosis was incorporated into other interventions, including Cognitive Behavior Therapy (CBT) to address his faulty beliefs and perfectionism, computerized biofeedback to enhance his psycho-physiological reactivity, and parent counseling to shift from their sense of helplessness to learning how to support their son’s own growing capacities to dampen strong emotions. He also kept a calendar to record his improving self-regulation skills, using a scale from 0 to 10 to indicate level of control over behavioral outbursts.

When designing a hypnotic or hypnosis trance experience, the clinician “utilizes” the child’s interests, capacities, and goals. J’s notable strengths included his passion and knowledge of baseball, his overall congenial personality and sportsmanship, and his motivation to gain self-control. Given his pronounced passion for baseball, the child and therapist made an agreement: He would teach her baseball terms and she would teach him “tricks” to improve his self-control and focus during baseball games. Accordingly, sports terminology, the self-calming rituals of his favorite star ball players, and his capacity to focus his attention were incorporated into therapeutic suggestions. In this regard, J. was asked to “teach” the clinician about sports slang and to tell some stories about key players. This language and information became “hooks” woven into suggestions during the hypnosis work.

For example, the phrase “on to the next one!” was introduced to J. as self-talk, an immediate inner response, to dissociate from any personal errors during the game and to sustain positive expectations. He also learned to “turn off the faucets” just before each practice and game, in order to control his past tearful responses and to regulate his intense emotional reactivity to mistakes. While he imagined playing a close game, he was taught to pair or link a calming breath with the touch of the ball when pitching, bat when hitting, and base when running.

He was also told a metaphoric story about a boy who decided it was time to no longer feel like a *loser* by being tricked by *curve balls* inside his head that were *stealing* his attention, focus and control. First, he found a *specialty coach* to work with on his *mental game* during his *spring training* and he found immediate *relief* with the *assist* of calming breaths. Second, he taught himself to *throw* away any *errors* that were *running around* in his head, in order to quickly *catch* on to practicing new *plays.* Third, he worked hard on *drills* to *line up* his new strategies in his mind. Before the end of his inner strength training, he *struck out* the old disappointment and frustration that had *fouled* up his *game*. He became a *champion* as a *triple threat* with his MVP—Most Valuable Playing—with full attention, focus, and self-control. *Watch him keeping his eyes on the ball, instead of bawling!*

This story was recorded and given to him, with instructions to listen to it daily as part of his ongoing practice doing self-hypnosis exercises to foster calmness and an enhanced capacity to modulate his emotions when coping with normal, every day “hassles”. In subsequent sessions, the clinician reviewed his progress calendar and added other hypnosis experiences for him to listen to daily.

Within a number of weeks, this child showed significantly enhanced self-regulation of his emotions and behavior, both on and off the field. He became an athlete who shared his self-control strategies with his teammates.

#### 7.1.2. CASE #2: Anger Management and Primary Nocturnal Enuresis

Almost 10-year-old “Z” was brought by his parents for “issues” of angry behavior and not wanting to do what he is told or asked, occurring exclusively at home. He has had no problems in school or in after-school programs, but acknowledged “issues” with stomping his feet, slamming doors, and defiantly refusing to do things he is “supposed” to do at home, either on his own or when asked.

The day before his first visit he had a public tantrum at a local store when his mother said he could not get the toy he wanted. As a “consequence” of his tantrum, he was “grounded” from using his electronic games for a week. His mother reported that she was surprised that he had spontaneously come to her to apologize that same evening before bed. He was surprised and proud when the clinician spontaneously gave him a “high 5!” for that spontaneous apology.

During the initial visit the clinician inquired what “issues” Z wanted to work on. He said he did not like the way he gets angry and was surprised when the clinician told him that “feeling angry is okay, everyone I ever met got angry or gets angry sometimes, and then they get un-angry. It is only okay or not okay *how* we get angry and *how* we act, not *if* we get angry. We are not allowed to hurt people or things when we get *angry*. The clinician noted Z’s spontaneous focused attention (spontaneous hypnotic trance) and complimented *all those other times that you might have gotten angry and did not.*

During the first visit the clinician identified Z’s strengths, including cooperation at school (i.e. no behavior problems or acting out there), great soccer skills, and being a “quick learner”.

Almost as an after-thought Z. mentioned that he had an “issue” with his bladder and he and his parents noted that he *never* wakes up in a dry bed. When asked about the *reason* for this, they said “because he sleeps too soundly, he’s a heavy, deep sleeper”. When he told the clinician that there was no problem with daytime wetting *ever*, the clinician purposely and dramatically showed pleasant surprise: “*really*? Well, that’s going to make this *much easier to solve* than I thought, we’ll work on this as soon as you know how to manage the *angry* feelings.” This “waking hypnotic suggestion” was planted as a seed for building upon at a future visit.

At the second visit, Z. was taught a hypnotic anger (or other feeling) management strategy called the “jettison technique”. Offering a “personal” example, the clinician gave a blank piece of paper to Z. and one for himself. Without formally mentioning hypnosis, the clinician said “Just listen and watch first. Then you’ll have a chance to *do* this too… Sometimes when I feel angry I just close my eyes or I look inside my mind with my eyes *open*—either way is fine—and I picture what *color and shape* angry is…. Let’s see, hmmmm, right now it’s a *red square*! Then, I imagine or pretend there is a faucet or drain pipe in the side of that square. I turn it on and the angry feeling runs out of my thinking (touching each next part while describing it) down the side of my neck, down my shoulder on my (either) arm and down and around the bend at the elbow and all the way down into the palm of my hand (holding palm upward) until all of the angry feeling has filled up my hand. Then I roll it up into a tight fist, take a deep breath and hold it, then as I blow out *I throw it away*!! (forcibly crumpling up the piece of paper as tight as possible and throwing it onto the floor!). At the end, I look *back* into my thinking to see the *color and shape of comfortable or relief or relaxed*…wow, now it’s a yellow circle, smiley face!! Okay, your turn!” He reproduced it, reporting different colors and shapes than the clinician’s. It was suggested that he have a pile of scrap paper in his room to use “as needed”.

At the next visit he and his parents reported considerably fewer tantrums and much more cooperation. He asked about getting *dry* at night. He was told quite directly that his brain and bladder *know each other very well,* that they work together great, and are in a good *pattern* of talking to each other, so much so that he does not wet his pants *even* if he has to “go really, really badly…” He agreed, and said that what was just described was exactly what he *does* every day all day except that he does not say it *out loud*.

Z. demonstrated another spontaneous hypnotic state while focusing carefully on a participatory drawing of genitourinary anatomy and physiology, capitalizing on his curiosity about how the body works, and his quick learning.

In the drawing, urine was depicted being sent from the kidneys (aka “pee factory”) to the bladder, shown as a “sack with a muscle around it” with a “gate” made of muscle that is closed most of the time, opening only upon signal from the “main computer”, *i.e*., the brain, when it is full. The drawing showed the brain and bladder connection, with bi-directional arrows communicating between the full bladder and the brain, keeping the gate “closed” until the right time and place.

Z. was taught that nighttime accidents are just that, *i.e.* that *his brain and bladder accidentally got into an accidental habit or pattern of not talking with each other at night*. Therefore, he has to remind them before he goes to sleep *because* he is going to be asleep. Since the bladder and brain have perfect communication all day long, they “are already friends and know how to talk to each other, so *all* you have to do is *remind them before you go to sleep.*”

Z. committed to giving instructions at bedtime to his brain and bladder “taking no longer than 5 min to do so”. “For example, you could say “Bladder, tonight let the brain know when you’re full. Brain, you have a choice: WHEN the bladder says it’s full, tell me to wake up, walk to the bathroom, open the gate, pee in the toilet, close the gate, flush , and go back to my *nice, warm, comfortable dry bed*, or, instead *tell the bladder gate to stay closed and locked all night until morning*, and *then* wake up happy and proud in a nice warm dry bed” With either choice, the bed is dry in the morning.

Z listened carefully. His parents and he agreed to discontinue using “diapers” (pull-ups) that he had worn—and soaked—nightly. Although they missed the scheduled appointment 2 weeks later, they returned in 4 weeks to report 29 out of 32 nights being totally *dry*, or 91% dryness. His parents were astounded, and he was very proud.

#### 7.1.3. Case #3: Fear of Shots and Embarrassing Warts

K. was a 10-year-old fifth grader who asked her parents to see the “special doctor” who helped her good friend get over daily tummy aches while at school when she was away from her Mom. K. wanted to get over her long-time fear of shots. She was also highly motivated to get rid of several large warts on her knees. She was tired of always wearing pants to cover them for fear of being teased.

The evaluation showed that her fear of injections met criteria for a Specific Phobia, given her excessive and developmentally inappropriate level of fear reactivity regarding the procedure. Her mother described K’s intense distress and panic whenever faced with this procedure in the past. She begged loudly for a delay, tried to hide in the corner of the exam room, cried and screamed in protest when the nurse approached her. Her screeches “No! No! No!” lasted until some minutes after the administration.

Given her panic symptoms of high psychophysiological and emotional reactivity, K. was taught diaphragmatic breathing (aka “belly breathing”) to activate the parasympathetic nervous system (aka the “calming control center, CCC”). She was urged to practice this new skill several times a day.

Because Exposure Therapy with Systematic Desensitization is the treatment of choice for phobias, K. was gradually exposed to the equipment (e.g., cleaning swab, syringe) and procedure for injections, until those items and “pretend” shot-giving no longer evoked activation of panic symptoms. In addition to this “*in vivo*” exposure, she was taught self-hypnosis to be used as repeated “imaginal exposure” that focused on her capacity for self-regulation of her body’s “false alarm” that had prompted such strong reactions with her emotions, thoughts, and behavior.

Given her gift for creative writing and love of biographies about strong-willed girls who became famous women, she was encouraged to “go to a special *safe* place where she was completely in charge and in control of herself”. She was invited to “just enjoy being *there* where she could also *now* turn on a special TV in her mind”. Suggestions were offered “to use your imagination to watch a Mastery Movie on the K Channel. Instead of watching the *old* disaster film of being out of control while getting a shot, this is a ‘*future* film’, you are the scriptwriter, the producer, the director, *and* the star of the show”.

She was encouraged to use her five senses to intensify the experience “*as if* you are really there: calmly and confidently walking into the waiting room, becoming even more relaxed and completely in control as you enter the exam room. Now notice how easily you deepen your sense of calmness by using your belly breathing and self-hypnosis when the nurse comes in to *give* the medicine *that keeps you healthy*. Pay attention to how you go so quickly to your Special Place so you can be more *there where* you’d like to be, there where you *don’t care* while the nurse *takes care* of the arm and the medicine. And, when it’s over, then you’ll be done, so when you’re ready you can now return to your usual alertness and awareness”.

K was also taught to use self-hypnosis to get rid of “those warts you don’t need anymore”. In each session, she experienced hypnosis so she could “use your imagination and creative mind to shrink yourself down in order to travel inside your body”. She had already been taught about the blood vessels underneath the warts. The suggestion was given to “decide for yourself your own special way to stop the blood supply from feeding the warts”. She heard a few ideas used by other children, such as a rope and lasso, a knife, a detour. Within a few moments her dominant handmade slashing gestures, as if she held a knife. Such ideomotor gestures are common during hypnosis.

K. practiced her self-hypnosis three times daily for two weeks, re-enacting the “scenes” elicited above. Upon return the warts were notably smaller, and she indicated readiness for the needed immunization. Two weeks later she returned again, wearing a lovely skirt, and proudly announced her successful experience with that immunization procedure. The warts were gone.

#### 7.1.4. CASE #4: Adolescent with Chronic Daily Headache (HA)

W was 15-2/12 at the time of her first visit. She was referred by one of her two pediatric neurologists for help with chronic, daily, severe headaches.

A detailed history was taken, revealing headaches since early childhood that increased in frequency, duration, and severity over time. There was a very strong family history of migraines. Over the years, she was evaluated by many physicians at various clinics and hospitals for the continuing severe headaches triggered by “lack of sleep/water, MSG, scents, physical activity, thinking, breathing, living in general.” Diagnostic testing for autonomic dysfunction revealed no positive results. Interventions included multiple medication trials, and a recent three-month intensive inpatient rehab program that involved a comprehensive and multi-modal approach: physical therapy, “group biofeedback”, relaxation including diaphragmatic breathing, listening to music, meditation, progressive relaxation, distraction, socialization with games, reading, movies, peers and positive self-talk, and an agreement to avoid opioids and daily pain medications. There was no mention of hypnosis.

W.’s academic history was marked by absence and numerous changes in locations and types of schooling.

During the initial visit, the typical *assessment* scale to measure pain severity scale was also used *therapeutically*. Using an agreed upon range of 0–12 (12 = worst imaginable HA, 0 = none), her average was eight. Surprised with the question: “What would you *prefer*?” she replied, “Four or less but I don’t think zero would be a good idea because I do feel I have this for a reason and it has enabled me to meet people.” When asked the last time it was *zero* she indicated “before it became daily 17 months earlier”.

The clinician gently corrected her by offering a *re-framing idea* that it is *zero* “whenever you are asleep” and in a purposely constructed “waking hypnotic suggestion”, the clinician said “So, *all* we have to do really is find out how to move the file in your brain called *no headache* from only at night to also when you’re awake”. As with most patients with chronic recurrent pain, the clinician introduced the possibility of change while simultaneously assessing motivation through the use of embedded suggestions with carefully selected wording in the following questions:

Will you miss the HA’s when they’re gone? ➔ “No.”
Is there anything good about the HA’s? ➔ “No”
Do you need them for anything? ➔ “No”
Do you think you have had them long enough? ➔ “Yes”

Given W.’s very chronic and severe headache history without relief despite multiple interventions, the clinician then set a therapeutic goal to *shift her focus* from over-investment in her pain to positive expectations for self-healing. W. agreed to keep a written HA diary, and was given a therapeutic suggestion “to track the reduced frequency and intensity. The clinician also asked W. if she “needed to find or know the “reason” for the HA if she could find a way for it to not bother her”, and she said she did NOT need to know the reason. *Of course had she said she did need to know the reason, additional and alternative clinical approaches would have been the appropriate next step.*

During the initial visit, multiple therapeutic seeds of “waking suggestions” were planted for her consideration, including (1) pain is pain, suffering is optional; (2) pain is a signal, nothing more, nothing less; and (3) when everything that can be done and should be done, has been done, there is no reason for pain to bother. At the end of this visit the clinician again evoked positive expectations when telling W. that he was absolutely sure he could *help her help herself*, that everyone whom he had ever met with a HA problem had gotten better without exception, and that she was invited to leave all past, present, and future headaches in the clinician’s file cabinet. She laughed.

At the second visit, training in hypnosis and self hypnosis began with viewing a couple of videos of youngsters both talking about and doing self-hypnosis. Induction (initiation) of hypnosis was easily accomplished with “favorite place imagery”. Suggestions for multisensory imagery along with progressive relaxation served to intensify (or “deepen”) the hypnotic experience. To amplify that self-hypnosis training emphasizes personal choice and self-control, therapeutic hypnotic suggestions for controlling HA were offered as a “menu” from which W could choose. For example, she was told “I don’t know how you will lower those HA’s that used to be there more, but somewhere in your inner mind you know how because you want to be and can be the boss of those HA’s instead of them begin the boss of you. I’d like to tell you some stories of how *other* young people got rid of *their* HA’s….I knew this one 12-year-old boy who had migraines and he used the idea of being on an elevator, so if he had a migraine that was a ’10’, he’d push the button to ride down to nine, and then the light would go off at 10 and on at nine, and then eight….and then . . . . . . six or five . . . . . and . . . then . . . . all the way to one or zero,” I met this one little girl who was only five; and she had a stomach-ache problem and she didn’t like elevators, so what *she* did in *her* imagination was imagine being on a water slide; and the 12 was at the top and the zero was at the bottom in the cool refreshing water, and whenever she had a tummy ache she would get on the water slide in her mind and zooooommmm down from 11-10-9-8-7-6-5-4-3-2-1-splash! A few years ago, I worked with a 17-year-old young woman, with migraine problems, who rode horses. To control her migraines she’d imagine riding on the beach with the horses’ hooves gradually erasing the zero–12 numbers in the sand. And, of course, when it got to zero it stayed there. I don’t know which of those stories you’ll use, or maybe some combination, or probably you’ve already come up with a couple in your own mind from your experiences. And, like everything, the more you practice, the better you’ll get. Whichever works best for you is the best for you.”

W. practiced self-hypnosis a couple of times each day as suggested. She improved gradually. She agreed to also keep a concurrent diary of “How am I dealing with it?” ratings, with zero = dealing with it best, and 12 = worst. W. soon reported much less use of medication for “severe” HA, and decrease of migraine prevention and anxiolytic medications.

By the middle of the following school year she was substantially improved. Nine months after the first visit, W. reported that her average HA rating was now down to 3.6 (from average of 8.5) and her “dealing with it” had improved 23%! to 2.3 With the intention of increasing her awareness of and empowering her own abilities the clinician asked W. “How did you DO that?” She replied “I am *stunned* it is *astonishing*… I never thought I could do it… you know, change my body with my mind. I did continue what you told me to do, so I imagine being at the cabin, and reading and then I swim in the lake and the closer I come back to shore the lower the number of my HA is and when I get to shore it’s fine!”

## 8. Contraindications and Caveats

While the foregoing cases are representative of common conditions *and* common responses to hypnotic interventions, it is essential to note that not everyone responds so positively and so quickly. Like any strategic therapeutic intervention, positive rapport is essential to good clinical work, and ongoing re-evaluation, flexibility, and engagement in brainstorming with patients and families are fundamental to ultimate progress, problem resolution, and healing.

As with any clinical intervention, treatment with clinical hypnosis is not without obstacles or roadblocks. As with any skill, rehearsal (“practice”) of self-hypnosis skills enhances competence, confidence, and positive outcomes whereas absence of regular rehearsal is more likely to result in slower and/or less positive results. We give “homework” assignments involving practice of self-hypnosis, counsel parents about their level of involvement *versus* interference, and encourage children and youth to come to appointments *with* their hypnosis and not only *for* it. Not unlike other therapies, however, inconsistent follow-up appointments and/or inconsistent attention to home rehearsal are potential contributors to less than desired outcomes.

For some children’s problems—as noted in the foregoing tables, hypnosis may be clearly the treatment of choice (e.g., enuresis, migraine) and have the best “track record” compared to what might otherwise have been considered “traditional” approaches to that problem. By contrast, for other problems/conditions (e.g., behavioral and mental health issues) hypnosis may be clearly more adjunctive, but nonetheless highly valued as an important ingredient in an overall management plan (e.g., with asthma or other chronic illnesses where learning self-hypnosis may not provide “cure” or resolution, but can/will contribute to reduced morbidity such as less medication needs, fewer days missed from school, better sleep, *etc.*) [2,30].

## 9. Contraindications

There are a few absolute contraindications to the utilization of hypnosis with children, and several more relative contraindications [2].

- Hypnosis should not be utilized for entertainment. As we have discussed and illustrated, hypnosis is a very effective clinical tool; and often may have powerful and dramatic positive effects on medical and mental health. We strongly advocate against the use of hypnosis for public entertainment as is seen commonly during high school graduation parties.
- Hypnosis should only be used by clinicians trained in the appropriate use of hypnosis and hypnotherapy and within the scope of their clinical practice. Thus, while hypnosis/hypnotherapy are very appropriate and useful adjuncts to help children with anxiety and/or discomfort during dental work, it would of course be unethical and inappropriate for a pediatrician or a psychologist who has learned hypnosis to start doing dental extractions! Of course it would be appropriate for those professionals to teach their clients how to use self-hypnosis WHEN they go to the dentist).

Analogously, though hypnosis can be a very helpful tool in treating posttraumatic stress disorder (PTSD), clinicians who learn hypnosis yet have no specialized training in treating children with PTSD should not start offering hypnosis for PTSD.

- Hypnosis should not be used to treat a condition for which there is already a fundamentally appropriate, acceptable, accessible, and effective treatment. One example might be strep throat. Or appendicitis. Of course, however, one might well learn self-hypnosis to help allay anxiety or discomfort associated with having a throat culture; or learn methods of control of anxiety, discomfort, and return of normal bodily functions pre- and post-operatively for an appendectomy.
- Hypnosis should be tailored to the developmental level and capacities of the individual child. Designing a hypnosis session to address clinical anxiety for a six-year-old would be very different for a nine-year-old. Typically, developing toddlers and those children with significantly impaired intellectual and language abilities and limited capacity for internal absorption would benefit from repetitive sensorimotor stimuli known to induce a calmer, steadier state of comfort, such as rhythmic rocking, patting, swaying, or music.

## 10. HOW?: Learning Hypnosis Skills

While this article is intended to be an overview of the value of clinical hypnosis in children, it is neither a “HOW to do it…” nor a manual or a written suggestion to imply that clinicians reading this article can now go to the office and begin using hypnosis. Quite the contrary, even the case illustrations noted carry the **notable *caveat*** that the involved experienced pediatric clinicians were very well trained in pediatric clinical hypnosis *before* they began to apply these skills in their clinical work.

It is our hope and intention that the foregoing will serve as a stimulus for further study, inquiry, and most importantly, for appropriate clinical skill development through training by licensed health care professionals. Pediatric-specific didactic presentations and experiential learning through supervised practice by licensed pediatric professionals are essential to the development of the expertise required to help children and families help themselves through the learning of self-hypnosis in fostering self-regulation abilities and other internal resources, as well as symptom reduction.

As briefly noted earlier, starting in 1987, a cadre of multidisciplinary faculty of licensed health care professionals began teaching what has become the longest-running pediatric clinical hypnosis workshop training in the United States, sponsored by the Society for Developmental and Behavioral Pediatrics (SDBP). Beyond those twenty-four years of workshops, the enduring and growing Faculty initiated the National Pediatric Hypnosis Training Institute (NPHTI = nifty!) in 2010 which continues to provide the tri-level pediatric hypnosis training workshops annually. Over the past 4 years over 400 child health professionals with graduate degrees have availed themselves of the NPHTI skill development workshops. These past participants, representing over forty states in the U.S.A. and seven foreign countries, include pediatricians, pediatric subspecialists (e.g., developmental-behavioral pediatricians, pediatric-trained pulmonologists, gastroenterologists, neurologists, oncologists, anesthesiologists), pediatric psychologists and child and adolescent psychiatrists, pediatric social workers, marriage and family therapists, Child Life Specialists, advanced practice pediatric nurses (e.g., clinical nurse specialists, pediatric nurse practitioners, and advanced practice RNs), and pediatric occupational therapists and physical therapists.

NPHTI is a rapidly growing professional organization open to inquiries from licensed professionals with advanced degrees and would be pleased to welcome qualified applicants to future training. (See www.nphti.com).

## References

[1] Mead M. (1949). Male and Female: A Study of the Sexes in a Changing World.

[2] Kohen D.P., Olness K.N. (2011). Hypnosis and Hypnotherapy with Children.

[3] Baldwin J.M. (1891). Suggestion in Infancy. Science.

[4] Erickson M.H. (1958). Pediatric Hypnotherapy. Am. J. Clin. Hypn..

[5] Wright M.E. (1960). Hypnosis and Child Therapy. Am. J. Clin. Hypn..

[6] Baumann F.W., Hinman F. (1974). Treatment of incontinent boys with non-obstructive disease. J. Urol..

[7] Baumann F.W. (1970). Hypnosis and the adolescent drug abuser. Am. J. Clin. Hypn..

[8] London P., Cooper L.M. (1969). Norms of hypnotic susceptibility in children. Dev. Psychol..

[9] Barber T.X., Calverley D.S. (1963). “Hypnotic-like” suggestibility in children. J. Abnorm. Soc. Psychol..

[10] Gardner G.G. (1978). Hypnotherapy in the management of childhood habit disorders. J. Pediatr..

[11] Olness K. (1975). The use of self-hypnosis in the treatment of childhood nocturnal enuresis. A report on forty patients. Clin. Pediatr..

[12] Zeltzer L.K., LeBaron S. (1982). Hypnosis and non-hypnotic techniques for reduction of pain and anxiety during painful procedures in children and adolescents with cancer. J. Pediatr..

[13] Zeltzer L.K., LeBaron S., Zeltzer P.M. (1984). The effectiveness of behavioral intervention for reducing nausea and vomiting in children and adolescents receiving chemotherapy. J. Clin. Oncol..

[14] LeBaron S., Hilgard J.R. (1984). Hypnotherapy of Pain in Children With Cancer.

[15] Olness K.N. (2014). Personal Communication.

[16] Gardner G.G., Olness K.N. (1981). Hypnosis and Hypnotherapy with Children.

[17] Olness K.N., Gardner G.G. (1988). Hypnosis and Hypnotherapy with Children.

[18] Olness K.N., Kohen D.P. (1996). Hypnosis and Hypnotherapy with Children.

[19] Olness K.N., Kohen D.P. (2001). Lehrbuch der Kinderhypnose und hypnotherapie.

[20] Olness K., Kohen D.P. (2006). Hypnose et Hypnotherapie chez l’enfant.

[21] Kohen D.P., Olness K.N., Colwell S., Heimel A. (1984). The use of relaxation/mental imagery (self-hypnosis) in the management of 505 pediatric behavioral encounters. J. Dev. Behav. Pediatr..

[22] Kuttner L. No Fears, No Tears (29 min). DVD. http://bookstore.cw.bc.ca.

[23] Kuttner L. No Fears, No Tears 13 Years Later: Children Coping with Pain (46 min). DVD. http://bookstore.cw.bc.ca.

[24] Kuttner L. (1988). Favourite stories. A hypnotic pain reduction technique for children in acute pain. Am. J. Clin. Hypn..

[25] Kuttner L. *Making Every Moment Count Documentary* (38 min); Co-production with The National Film Board of Canada, 2003. www.nfb.ca.

[26] Olness K.N., Culbert T.C., Uden D. (1989). Self-regulation of salivary immunoglobulin A by children. Pediatrics.

[27] Sugarman L.I. (2006). Hypnosis in Pediatric Practice: Imaginative Medicine in Action.

[28] Thomson L. (2005). Harry Hypno-Potamus: Metaphorical Tales for Pediatric Problems.

[29] Thomson L. (2009). Harry Hypno-Potamus: More Metaphorical Tales for Pediatric Problems.

[30] Sugarman L.I., Wester W.C. (2014). Therapeutic Hypnosis with Children and Adolescents.

[31] National Pediatric Hypnosis Training Institute. www.nphti.com.

[32] Trenkle B. (2014). Kindertagung History—Heidelberg, Germany. Personal Communication.

[33] Kohen D.P. (1995). Relaxation/Mental imagery (self-hypnosis) for childhood asthma: Behavioral outcomes in a prospective, controlled study. Hypnos.

[34] Kohen D.P., Wynne E. (1997). Applying hypnosis in a preschool family asthma education program: Uses of storytelling, imagery and relaxation. Am. J. Clin. Hypn..

[35] Kohen D.P., Botts P. (1987). Relaxation-imagery (self-hypnosis) in Tourette syndrome: Experience with four children. Am. J. Clin. Hypn..

[36] Lynn S.J., Rhue J.W. (1991). Theories of Hypnosis: Current Models and Perspectives.

[37] The Society of Psychological Hypnosis, Division 30 of the American Psychological Association. http://psychologicalhypnosis.com/info/.

[38] Sugarman L.I. Re-Thinking Hypnosis/Refining Utilization. Proceedings of Intermediate Pediatric Hypnosis Workshop—National Pediatric Hypnosis Training Institute.

[39] Rainville P., Carrier B., Hoffbauer R.K., Bushnell M.C., Duncan G.H. (2001). Dissociation of sensory and affective dimensions of pain using hypnotic modulation. Pain.

[40] Raz A. (2011). Does neuroimaging of suggestion elucidate hypnotic trance?. Int. J. Clin. Exp. Hypn..

[41] Wagstaff G.F. (2014). On the centrality of the concept of an altered state to definitions of hypnosis. J. Mind-Body Regul..

[42] Kaiser P. (2011). Chlldhood Anxiety, Worry, and Fear: Individualizing Hypnosis Goals and Suggestions for Self-Regulation. Am. J. Clin. Hypn..

[43] Berger A. (2011). Self-Regulation: Brain, Cognition, and Development.

[44] Hall H. Resources for Future Training. Presented at National Pediatric Hypnosis Training Institute Introductory Workshop.

[45] Kohen D.P., Culbert T.P., Olness K. (2010). A pediatric perspective on mind-body medicine. Integrative Pediatrics.

[46] Yapko M.D. (2012). Trancework: An Introduction to the Practice of Clinical Hypnosis.

[47] Benson H. (1990). The Relaxation Response.

[48] Kohen D.P. (1996). Interview and demonstration of Relaxation and Mental Imagery for an 11 y.o. Boy (John H.) with Asthma and Anxiety.

[49] Olness K., MacDonald J.T., Uden D.L. (1987). Comparison of self-hypnosis and propranolol in the treatment of juvenile classic migraine. Pediatrics.

[50] Dikel W., Olness K. (1980). Self-hypnosis, biofeedback, and voluntary peripheral temperature control in children. Pediatrics.

[51] Olness K.N., Conroy M. (1985). Voluntary control of transcutaneous pO2 by children. Int. J. Clin. Exp. Hypn..

[52] Kohen D.P., Ondich S. (2004). Children’s self-regulation of cardiovascular function with relaxation mental imagery (self-hypnosis): Report of a controlled study. Hypnos: J. Eur. Soc. Hypn. Psychother. Psychosom. Med..

[53] Lee L.H., Olness K.N. (1996). Effects of self-induced mental imagery on autonomic reactivity in children. J. Dev. Behav. Pediatr..

[54] Anbar R.D. (2000). Self-hypnosis for patients with cystic fibrosis. Pediatr. Pulm..

[55] Olness K.N. (1981). Imagery (self-hypnosis) as adjunctive therapy in childhood cancer: Clinical experience with 25 patients. Am. J. Pediatr. Hem./Onc..

[56] Jacknow D.S., Tschann J.M., Link M.P., Boyce W.T. (1994). Hypnosis in the prevention of chemotherapy—Related nausea and vomiting in children: A prospective study. J. Dev. Behav. Pediatr..

[57] Kohen D.P., Zajac R. (2007). Self-hypnosis training for headaches in children and adolescents. J. Pediatr..

[58] Kohen D.P. (2010). Long-term follow-up of self-hypnosis training for recurrent headaches: What the children say. Int. J. Clin. Exp. Hypn..

[59] Sugarman L.I., Garrison B.L., Williford K.L. (2013). Symptoms as solutions: Hypnosis and biofeedback for autonomic regulation in autism spectrum disorders. Am. J. Clin. Hypn..

[60] Kohen D.P. (1996). Management of trichotillomania with relaxation/mental imagery (self-hypnosis): Experience with five children. J. Dev. Behav. Pediatr..

[61] Thomson L. (2002). Hypnosis for habit disorders. Helping children help themselves. Adv. Nurse Pract..

[62] Gottsegen D.N. (2003). Curing bedwetting on the spot: A review of one session cures. Clin. Pediatr. (Phila).

[63] Anbar R.D., Hall H.R. (2004). Childhood habit cough treated with self-hypnosis. J. Pediatr..

[64] Shenefelt P.D. (2004). Using hypnosis to facilitate resolution of psychogenic excoriations in acne excoriee. Am. J. Clin. Hypn..

[65] Anbar R.D. (2007). User friendly hypnosis as an adjunct for treatment of habit cough: A case report. Am. J. Clin. Hypn..

[66] Cyr L.R., Culbert T., Kaiser P. (2003). Helping children with stress and anxiety: An integrative medicine approach. Biofeedback.

[67] Tschann J., Kaiser P., Boyce W.T., Chesney M.A., Alkon-Leonard A. (1996). Resilience and vulnerability among preschoolers: Family functioning, temperament, and behavior problems. J. Child Adol. Psychiatry.

[68] Culbert T., Richtsmeier-Cyr L., Culbert T., Richtsmeier-Cyr L. (2005). Pediatric Mind/Body Medicine: The Hypnosis/Biofeedback Interface. (Entire Issue). Biofeedback Newsmagazine of the AAPB.

[69] Olness K., Carey W., Crocker A., Elias E., Feldman H., Coleman W. (2009). Self control and regulation. Developmental Behavioral Pediatrics.

[70] Yapko M. (2011). Mindfulness and Hypnosis: The Power of Suggestion to Transform Experience.

[71] Kohen D.P. (2011). Chronic daily headache: Helping adolescents help themselves with self-hypnosis. Am. J. Clin. Hypn..

[72] Labaw W.L. (1975). Auto-hypnosis in hemophilia. Haematologia.

[73] Anbar R.D. (2003). Self-hypnosis for anxiety associated with severe asthma: A case report. BMC Pediatr..

[74] Hackman R.M., Stern J.S., Gershwin M.E. (2000). Hypnosis and asthma: A critical review. J. Asthma..

[75] Culbert T., Kajander R., Kohen D., Reaney J. (1996). Hypnobehavioral approaches for school-age children with dysphagia and food aversion. J. Dev. Behav. Pediatr..

[76] Gottsegen D. (2011). Hypnosis for functional abdominal pain. Am. J. Clin. Hypn..

[77] Kuttner L., Chambers C.T., Hardial J., Israel D.M., Jacobsen K., Evans K. (2006). A randomized trial of yoga for adolescents with irritable bowel syndrome. Pain Res. Manag..

[78] Vlieger A.M., Menko-Frankenhuis C., Wolfkamp S.C.S. (2007). Hypnotherapy for children with functional abdominal pain or Irritable Bowel Syndrome: A randomized controlled trial. Gastroenterology.

[79] Pendergrast R.A. (2003). Imagine Something Different. Arch. Pediatr. Adolesc. Med..

[80] Kohen D.P., Lankton S.R., Zeig J.K. (1995). Ericksonian communication and hypnotic strategies in the management of tics and Tourette syndrome in children and adolescents with Tourette syndrome. Difficult Contexts for Therapy—Ericksonian Monographs.

[81] Kuttner L., Goldman A., Hain R., Liben S. (2006). Pain—An integrative approach. Oxford Textbook of Palliative Care for Children.

[82] Berberich F.R., Landman Z. (2009). Reducing immunization discomfort in 4- to 6-year-old children: A randomized clinical trial. Pediatrics.

[83] Myers C.D., Bergman J., Zeltzer L.K., Kreitler S., Arush M.B. (2004). Complementary and alternative medicine use in children with cancer. Psychosocial Aspects of Pediatric Oncology.

[84] Zeltzer L.K., Dolgin M.J., LeBaron S., LeBaron C. (1991). A randomized, controlled study of behavioral intervention for chemotherapy distress in children with cancer. Pediatrics.

[85] Anbar R.D. (2001). Self-hypnosis for management of chronic dyspnea in pediatric patients. Pediatrics.

[86] Antich J.L.S. (1967). The use of hypnosis in pediatric anesthesia. J. Am. Soc. Psychosom. Dent. Med..

[87] Bernstein N.R. (1965). Observations on the use of hypnosis with burned children on a pediatric ward. Int. J. Clin. Exp. Hypn..

[88] Butler L.D., Symons B.K., Henderson S.L., Shortliffe L.D., Spiegel D. (2005). Hypnosis Reduces Distress and Duration of an Invasive Medical Procedure for Children. Pediatrics.

[89] French G.M., Painter E.C., Coury D.L. (1994). Blowing away shot pain: A technique for pain management during immunization. Pediatrics.

[90] Kohen D.P. (1996). Applications of relaxation/mental imagery (self-hypnosis) in pediatric emergencies. Int. J. Clin. Exp. Hypn..

[91] Kuttner L. (2010). A Child in Pain: How to Help: What Health Professionals Can Do to Help Crown.

[92] Tsao J.C.I., Meldrum M., Kim S.C., Jacob M.C. (2007). Treatment preferences for CAM in pediatric chronic pain patients. Evid.-Based Complement. Altern. Med..

[93] Uman L.S., Chambers C.T., McGrath P.J., Kisely S.A. (2008). A systematic review of randomized controlled trials examining psychological interventions for needle-related procedural pain and distress in children and adolescents: An abbreviated Cochrane Review. J. Pediatr. Psychol..

[94] Kaiser P. (2014). Childhood anxiety and psychophysiological reactivity: Hypnosis to build discrimination and self-regulation skills. Am. J. Clin. Hypn..

[95] Spies G. (1979). Desensitization of test anxiety: Hypnosis compared with biofeedback. Am. J. Clin. Hypn..

[96] Kuttner L., Friedrichsdorf S.J., Sugarman L.I., Wester W.C. (2014). Hypnosis and palliative care In Therapeutic Hypnosis with Children and Adolescents.

[97] Golden W. (2012). Cognitive Hypnotherapy for Anxiety Disorders. Am. J. Clin. Hypn..

[98] Kerns C.M., Read K.L., Klugman J., Kendall P.C. (2013). Cognitive-behavioral therapy for youth with social anxiety: Differential short and long-term treatment outcomes. J. Anxiety Disord..

[99] Yapko M.D. (2001). Treating Depression With Hypnosis: Integrating Cognitive-Behavioral and Strategic Approaches.

[100] Yapko M.D. (2006). Applying Hypnosis in Treating Depression: Innovations in Clinical Practice.

[101] Kohen D.P., Murray K., Yapko M.D. (2006). Depression in Children and Youth: Applications of Hypnosis to Help Young People Help Themselves. Applying Hypnosis in Treating Depression: Innovations in Clinical Practice.

[102] Kohen D.P., Sugarman L.I., Wester W.C. (2014). Depression. Therapeutic Hypnosis with Children and Adolescents.

[103] Culbert T., Kajander R. (2007). Be the Boss of Your Sleep: Self-Care for Kids.

[104] Kuttner L., Brown D.C. (2009). Treating pain, anxiety and sleep disorders with children and adolescents. Advances in the Use of Hypnosis in Medicine, Dentistry, Pain Prevention and Management.

[105] Kohen D.P., Mahowald M.W., Rosen G.R. (1992). Sleep-terror disorder in children: The role of self-hypnosis in management. Am. J. Clin. Hypn..

[106] Stanton H.E. (1989). Hypnotic relaxation and the reduction of sleep onset insomnia. Int. J. Psychosom..

[107] Zeltzer L.K., Schlank C. (2005). Conquering your Child’s Chronic Pain.

[108] Gardner G.G. (1976). Childhood, death and human dignity: Hypnotherapy for David. Int J. Clin. Exp. Hypn..

[109] Gulewitsch M., Muller J., Hautzinger M., Schlarb A.A. (2013). Brief hypnotherapeutic-behavioral intervention for functional abdominal pain and irritable bowel syndrome in childhood: A randomized controlled trial. Eur. J. Pediatr..

[110] Schlarb A.A., Liddle C.C., Hautzinger M. (2011). JuSt—A multimodal treatment program for adolescents with insomnia. Pilot study. Nat. Sci. Sleep..

[111] Lifshitz M., Cusumano E.P., Raz A. (2013). Hypnosis as neurophenomenology. Front. Hum. Neurosci..

[112] Curtis S., Wingert A., Ali S. (2012). *The Cochrane Library* and Procedural Pain in Children: An Overview of Reviews. Evid.-Based Child Health: A Cochrane Rev. J..

[113] Gold J.I., Kant A.J., Belmont K.A., Butler L.D. (2007). Practitioner review: Clinical applications of pediatric hypnosis. J. Child Psychol. Psychiatry.

[114] Landier W., Tse A. (2010). Use of Complementary and Alternative Medical Interventions for the Managaement of Procedure-Related Pain, Anxiety, and Distress in Pediatric Oncology: An Integrative Review. J. Pediatr. Nurs..

[115] McClafferty H. (2011). Complementary, Holistic, and Integrative Medicine: Mind-Body Medicine. Pediatr. Rev..

[116] Richardson J., Smith J., McCall G., Pilkington J. (2006). Hypnosis for Procedure-Related Pain and Distress in Pediatric Cancer Patients: A Systematic Review of Effectiveness and Methodology Related to Hypnosis Interventions. J. Pain Symp. Manag..

[117] Schnur J.B., Kafer I., Marcus C., Montgomery G.H. (2008). Hypnosis to Manage Distress Related To Medical Procedures: A Meta-Analysis. Contemp Hypn..

[118] Uman L.S., Birnie K.A., Noel M., Parker J.A., Chambers C.T., McGrath P.J., Kisely S.R. (2013). Psychological interventions for needle-related procedural pain and distress in children and adolescents. Cochrane Database Syst. Rev..

